# Essential Concepts in Artificial Intelligence: A Guide for Pediatric Providers

**DOI:** 10.3390/children12101386

**Published:** 2025-10-14

**Authors:** Laura Elena Mendoza Bolivar, Michael Satzer

**Affiliations:** 1 The Children’s Hospital at Montefiore, Bronx, NY 10467, USA; 2 Anne and Robert H. Lurie Children’s Hospital of Chicago, Chicago, IL 60611, USA; msatzer@luriechildrens.org

**Keywords:** artificial intelligence, machine learning, deep learning, pediatric cardiology, congenital heart disease

## Abstract

Artificial intelligence (AI) has exploded in public awareness over recent years and is already beginning to reshape the health care sector. Yet, even as AI becomes more prevalent, it remains a mystery to many providers who lack hands-on exposure during their training or on the job. Intended for medical professionals, this article defines essential concepts in AI interspersed with illustrations of how such concepts may be applied within cardiology and radiology—fields that have garnered the most approved medical AI applications to date. No experience in the field of AI is requisite before reading. To assist providers encountering novel machine learning tools, we also present an AI model checklist to empower critical assessment. We finally discuss hurdles in the path of developing pediatric AI tools—including challenges distinct from the adult setting—and discuss potential solutions, including various methods of multisite collaboration. This article aims to increase the engagement of health care professionals who may encounter AI models in practice or who seek to become involved in AI development themselves. We encourage the reader the freedom to either peruse this article in its entirety or to reference specific concepts individually. Terminology central to machine learning is emphasized in bold.

## 1. Fundamental AI Concepts

### 1.1. What Is Artificial Intelligence?

**Figure 1:**
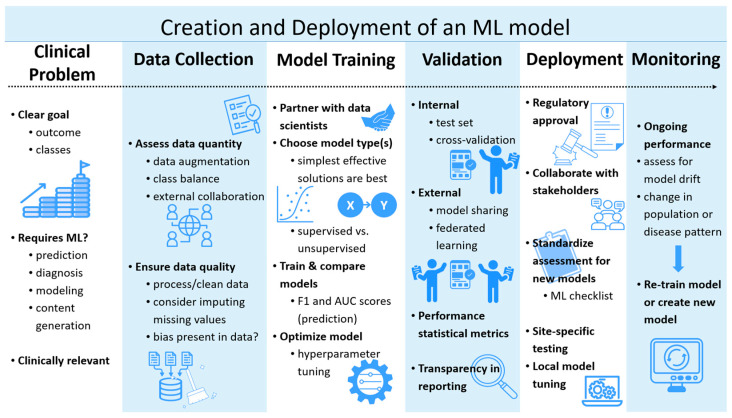
Pipeline illustration of the current creation and deployment of a machine learning model. ML: Machine Learning.

AI is the field of computer science that creates tools mimicking human intelligence by attempting to learn, analyze, and develop conclusions [1,2,3]. AI-based tools have helped to streamline healthcare systems and support clinical decision making since 1960. Studies have shown the capacity for AI algorithms to improve the accuracy of medical diagnosis and aid in clinical examination, risk stratification, management, and health maintenance [1,3,4]. The primary aims of AI in the medical field include task automation, disease detection and diagnosis, early prediction of adverse patient events, assistance with decision making, and simulation of complex systems (Figure 1).

### 1.2. What Is Machine Learning?

**Figure 2:**
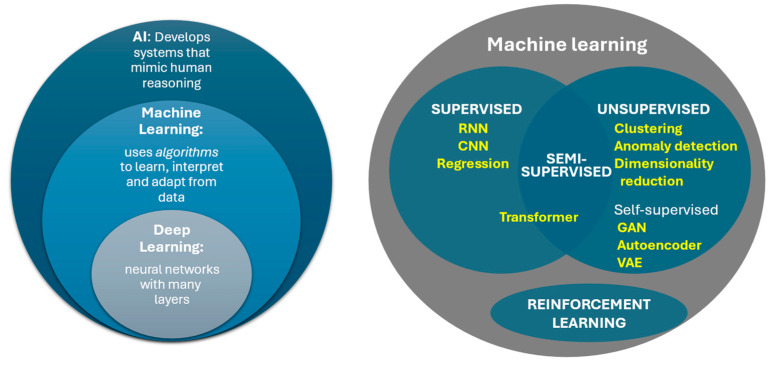
Relationship between basic concepts of AI. RNN: Recurrent Neural Network; CNN: Convoluted Neural Network; GAN: Generative Adversarial Network; VAE: Variational Autoencoder.

Machine learning (ML) is a branch of AI that uses algorithms to learn, interpret, and adapt from data (Figure 2). These algorithms generate **models**, sophisticated mathematical functions trained on an initial set of data to respond to new, unforeseen data [3]. Models do not have to be specifically programmed for every scenario, as they are uniquely capable of responding to unforeseen data through pattern recognition. ML models identify potentially non-intuitive and often non-linear relationships between the input (e.g., an electrocardiogram/ECG) and output (e.g., diagnosis of heart block). The “shape” of the input is arbitrary. It may be one-dimensional (e.g., sound recording from a stethoscope), two-dimensional (e.g., a still echocardiogram image or X-ray), or higher-dimensional (e.g., cardiac MRI data set). These models generalize from a large volume of examples to make predictions, categorize data, detect anomalies (atypical data points), or even generate new data such as text or images.

**Training** machine learning models occurs typically through one of the following paradigms of machine learning:

- **Supervised learning** models are trained with **labeled data** (e.g., ECGs tied to a specific diagnosis like supraventricular tachycardia or hypertrophic cardiomyopathy). The labels are established from a **ground truth**—something assumed to be correct from expert interpretation or another testing modality (e.g., echo or MRI). Another term for “label” in machine learning is **class**, a specific category that a model typically wants to predict, e.g., heart rhythms from an ECG or congenital heart disease types from an echo. Models “learn” fundamental patterns in the data that point toward the labeled outcomes. This type of training often comes at a significant cost of human time investment, due to the demand for large volumes of data accurately labeled by medical specialists.
- **Unsupervised learning** attempts to find patterns or relationships between the variables in a set of **unlabeled data**. Examples include clustering (grouping data points by similarity) and anomaly detection (identification of abnormal data points). Anomaly detection proves particularly useful in cases where the abnormality of interest is rare, potentially applicable to many pediatric conditions such as congenital, genetic, and metabolic abnormalities [5]. Scarcity of data labeled with a given disease hinders the development of supervised learning models. Another technique in unsupervised learning is the simplification of data sets with many variables (i.e., to reduce the number of dimensions of the input data) while still identifying **features** (essential aspects of a phenomenon from which a model learns and predicts). The output of these **dimensionality reduction** models may in turn become input to train other ML models, allowing the downstream model to be less complex and demand less computational resources.
- While technically a subset of unsupervised learning, **self-supervised learning** represents a powerful type of ML that also uses unlabeled data. This method recognizes inherent classes of data types and may even generate pseudo-labels for these groups. Moreover, self-supervised methods of pre-training may dramatically accelerate the development of a successful complex model. This involves the addition of an initial step called a pretext task, which is a challenge given while pre-training (preparing the basis of) a model for the benefit of learning rather than the task itself. One could analogize such preparation as AI grade school, preparing the model for higher education. Pretext task goals differ from the intended final goal. For image recognition, these may include filling in missing parts of an image or correctly rotating an image to its normal orientation. Language model pretext tasks might include predicting the network of an incomplete sentence. These challenges teach models the fundamentals of the subject of the data. The pre-trained model then goes on to train toward the final goal. Examples of models that may use self-supervised learning include autoencoders, computer vision, generative adversarial networks (GANs), and transformers—the last of which includes most large language models. Indeed, the “GPT” of the popularized chatbot ChatGPT-5 stands for “generative pre-trained transformer” [6].
- **Semi-supervised learning** is—in a sense—a hybrid approach between supervised and unsupervised learning, where a small portion of data is manually labeled. These models may help assign classifications to unlabeled data or learn implicit features from unlabeled data to later train more successfully with limited quantities of labeled data.
- **Reinforcement learning** takes continuous environmental feedback to optimize decision making. Most reinforcement learning uses a Markov decision process that operates via what is called an “agent” that takes actions based on the state of its environment, which result in an outcome and an associated reward signal. The outcome of the changed environment becomes the new input and the process is repeated. For example, an algorithm (agent) meant to suggest optimal drug dosing (action) can continuously learn from patient outcomes (reward signal) and optimize future recommendations. Reinforcement learning can occur in real time (online) or retrospectively (offline) [1,4].

### 1.3. What Is Deep Learning?

Deep learning is a subtype of machine learning in which models are composed of **neural networks**, constructed of multiple layers of artificial “neurons” or interconnected nodes that mimic the structure of a brain. The multiple internal layers are what makes it “deep.” Data is initially input to the first layer. Successive layers sequentially apply mathematical transformations to the output of prior layers. The exact operations of the transformations are adjusted in the training phase. Typically, each individual neuron sums each input value multiplied by a number (**weight**) to which another value is added (**bias**), with the resulting value passed through a simple function (**activation function**). Once trained, the weights and biases of the model constitute its **parameters**. The activation function is typically non-linear—a key feature that helps deep learning to model complex phenomena. The most basic neural network is completely fully connected, where every neuron in a layer is connected to (i.e., receives input from) each output from the prior layer. This is computationally inefficient, so most models only employ a limited amount of fully connected layers. Training may occur through any of the methods described above in Section 1.2 (Figure 2). Most artificial neural networks trained with supervised learning methodology use a process called backpropagation. This strategy progressively and repeatedly updates the model parameters (weights, biases) by small amounts based on how much each parameter seems to contribute to the error of the model. Each round of training on the full training data set with updates to the parameters is termed an epoch [1,4]. A fully trained model has values of its parameters optimized to the desired output of the model.

**Figure 3:**
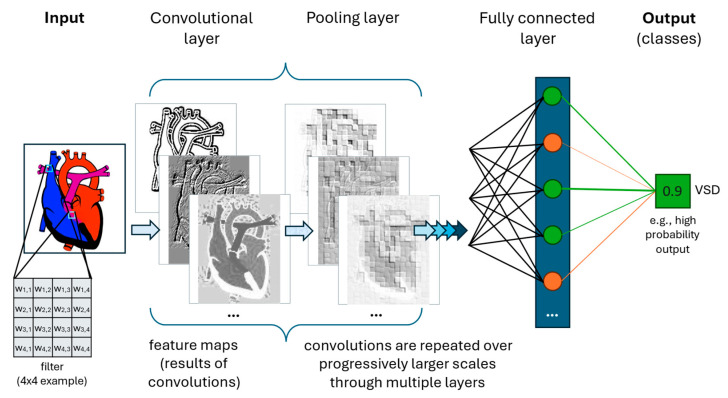
Simplified cartoon illustration of a single class predictive Convolutional Neural Network. Convolutional layers perform structured non-linear transformations. Multiple unique filters are employed per convolutional layer, with three resulting feature maps illustrated here. A pooling layer often follows convolutional layer(s), reducing the output size by summarizing localized information. Fully connected layer(s) are often added at the end of a CNN to reorganize the data from 2-dimensional (spatial) to 1-dimensional (linear), which facilitates the final steps in classification. The output is one or more outcomes (classes), here being the presence of a ventricular septal defect (VSD) based on a cardiac image.

**Convoluted Neural Networks (CNNs)** are a further subset of deep learning typically utilizing training through supervised learning. Classical CNNs expect input data to be structured like a grid, where data is contiguous in space, time, sequence, and/or another dimension—such as in a single echo image (spatially structured) or ECG (spatially and temporally structured). Convolutions use **filters** (also known as., kernels), which are small matrices used to extract **features** of the data. In each convolutional layer of the neural network, these filters are moved across the entire input data (e.g., all filters in the first layer scan every pixel of an image at least once). The output of a convolutional layer is a linear algebra operation (the summed element-wise product of the filter with the input data, plus a bias value), which is then run through an activation function—just as described above. Many different filters are used for each layer. Multiple convolutional layers progressively identify higher-order features across different scales. When interpretating an X-ray, for example, the first layers might be recognizing lines at different angles, middle layers identifying larger shapes, and later layers differentiating tissues or identifying abnormalities (e.g., enlarged heart or pneumonia). CNNs exhibit significantly better efficiency (less computational burden) and generalization (ability to interpret new data) than fully connected neural networks (Figure 3) [7]. CNNs in pediatric cardiology have shown, for example, the ability to enhance electrocardiogram (ECG) interpretation. Studies have demonstrated automated detection of ventricular dysfunction, risk for sudden death in congenital heart disease, and long QT syndrome, among other clinically significant diagnoses simply based on ECG [8,9,10]. For example, Mayourian et al. recently developed a CNN to detect LV dysfunction on standard 12-lead EKG with high accuracy (NPV 99%, AUROC 94% on external validation), supporting generalizability of the model [8]. Conventional algorithms have had poor success or been considered unfeasible toward such aims [8,9,10].

**Encoders** and **Decoders** are components of deep learning that transform data through compression (encoder) or decompression (decoder). The product of an encoder is a compressed, digitally smaller representation of the input data containing its intrinsic features occurring through a process called dimensional reduction. The work of encoders can be thought of as ignoring improbable states of the input. While a radiologist may diagnose many different findings on a chest X-ray, they would never look at a picture of a bird or a train and interpret it as an X-ray. Fundamentally, chest X-rays have characteristics that are less complex than any conceivable image of the same size. Encoders project the input data into **latent space**, a smaller “universe” into which the information fits. In the prior example, this would be “chest X-ray space.” Decoders can take a point from latent space and then reconstruct the higher-dimensional data (e.g., image) or something like it. Pairing an encoder with a decoder produces an **autoencoder**, a model that trains via unsupervised learning to reconstruct the original image from a smaller representation [11]. One notable variety, called a **Variational Autoencoder (VAE)**, forces the data in the latent space to be organized (normally distributed). This results in meaningful organization of this space. VAEs are thus excellent at generating new data points that are realistic, such as for **data augmentation** or for **imputation**. Data augmentation is an artificial expansion of training data useful when data sets are small or lack sufficient examples (e.g., cases of a rare disease). Imputation is filling in missing values—or even missing parts of an image. For example, if the input of a model is health record data from intensive care patients—but individual patients are missing specific values like lab tests—imputation may generate reasonable values for the missing test results to allow a model to be trained. VAEs and other autoencoders also excel at **anomaly detection**, or the recognition of abnormal examples that do not fit the “mold” of most of the data. Anomaly detection has demonstrated, for example, the ability to detect abnormal chest X-rays when trained on only normal radiographs [12]. Other benefits of autoencoders include **noise reduction** in images/data and generation of images or clinical scenarios for medical education [11,13].

Noise reduction through ML has the potential not only to improve image quality for interpretation, but also to reduce image acquisition time and thus exposure to ionizing radiation and/or anesthesia. Patients with congenital heart disease often require repeated chest imaging (e.g., X-ray, CT, fluoroscopy), accumulating radiation exposure that poses significant long-term risk of malignancy. Image artifact due to movement during cross-sectional imaging may lead to poor results or repeat imaging. Current techniques, such as those described by Schicchi et al. utilize approaches at the time of acquisition—dual-source dual-energy CT, enabling significant reduction in image acquisition time, with total radiation dose less than ≈1 mSv (equivalent to ≈10 chest X-rays), and CT contrast volume [14]. Even these advanced techniques could be further augmented in the post-acquisition stage by deep learning-based reconstruction, potentially the next paradigm shift to increase the clinical utility of imaging results and reduce exposure to X-ray and contrast agents [15].

The collective term for models such as encoder-decoders that create new content is **generative AI** (gen AI). Another example of this is a **generative adversarial network (GAN)**, which uses unsupervised training to generate simulated data based on real world data. GANs train via competitive (“adversarial”) neural network components: first, a generator network (often a trained decoder module of an autoencoder) produces new data in as similar a style as possible to the real data; and second, a discriminator network tries to identify correctly when an example is real or “fake” data. These components train together, competing until the data from the generator is indistinguishable from the style of the real data (e.g., creating simulated cardiac MRI images based on real ones). GANs can create images for data augmentation (see above) and have also been useful for image noise reduction. While a traditional GAN uses random noise as input to generate new images, variants may use modifiers or other images as source material. For example, a GAN model might produce an MRI-like image using CT or echocardiography images as a starting point. Specific to the medical space, MedGAN is a term used for a GAN able to generate synthetic medical data for the purposes of medical data augmentation, assistance with medical imaging tasks, or in silico drug development research [1].

**Recurrent Neural Networks (RNNs)** are a type of neural network that operate on sequential data and use feedback loops to “remember” aspects of preceding information and thus maintain awareness of context [1]. A common RNN subtype is Long Short-Term Memory (LSTM). Unlike many other neural networks, RNNs can accept sequentially input data of differing sizes. RNNs led to significant advances in **natural language processing (NLPs)** that interprets, classifies, translates, and/or generates human language [16]. Large Language Models (LLMs) are ML models that leverage (unsurprisingly) large data sets to accomplish NLP tests.

More recently, a more advanced neural network architecture called the **transformer** has supplanted RNNs in Large Language Models and underpins the current generation of chat-bots and translation applications. Like RNNs, transformers may take inputs of arbitrary length. More critical to their incredible performance is a technique called **self-attention**, which weighs the relative importance of the input components in addition to the connection between these components. This is most easily understood in the context of language: some words carry more importance than others, and a word’s meaning is contextually dependent on those around it. These techniques may easily translate into medical applications, including medical imaging, such as identification of radiographic abnormalities [1,17]. Transformers typically pre-train on massive datasets via semi-supervised learning, with further specialization of the models on smaller labeled data using supervised learning. Further development of medically specific LLMs may assist providers in summarizing patient history, documenting patient encounters, and extracting data from electronic health record (EHR) text for research. Recently, many medical and technology companies have released LLMs to assist with clinical decision making and research. Examples include Med-PaLM, BioGPT, PubMedGPT, Semantic Scholar, and many others [18,19,20]. Unfortunately, while LLMs do perform well when evaluated on various sectors of medicine, their accuracy falls short of 100% and may in cases provide irrelevant or misleading answers. The authors caution against the use of these tools in their current state for patient management without proper clinical judgement and fact-checking. However, LLMs have already shown promise in helping relieve the burden of clinical work. Providers may already be aware of LLMs integrated into medical systems, such as for automated note preparation, clinical summarizing, and suggesting responses to patient messages. Beyond use in language, transformers have also, for example, shown capability to predict DNA function and protein structure solely based on nucleic acid and amino acid sequences, respectively [21,22,23].

### 1.4. What Are Some Examples of Shallow Learning?

Despite the pejorative name, shallow learning AI techniques still retain merit. The most basic models are familiar to readers: linear and logistic regression. Slightly more “complex” are non-linear models, such as decision trees, random forest, k-nearest neighbor, K-means clustering, and support vector machine. **Decision trees** use a branching flowchart optimized to help make decisions. Starting from a single point (node), a series of dichotomous questions sequentially evaluates input variables to guide a path through the branching tree. At the end of each path lies an outcome or recommendation. **Random forests** use multiple decision trees constructed with randomized subsets of the data. Averaging of the individual trees’ outputs guides the model result. Random forests may improve accuracy and reduce overfitting compared to single decision trees (see Section 2 for a description of overfitting). **K-nearest neighbor** (KNN) is a supervised method for either classification or regression. It relies on the inherent relationship between data points and does not make assumptions about how these examples might be distributed. KNNs may be useful in classification. While akin to KNN in grouping data points, **K means clustering** is an example of unsupervised learning. It attempts to divide the data into K number of groups, each containing similar data points. Clustering is useful within medicine to identify patient subgroups, for example, and potentially the target therapy based on similarity in underlying disease phenotype. A **support vector machine** (SVM) seeks to separate two classes (outcomes) of data by drawing linear boundaries (hyperplanes) between examples of each class. This is yet another type of classification model. Points are plotted in a space with an arbitrary number of dimensions determined by the number of input variables. In some cases, SVMs transform the input data (via a kernel function), increasing the dimensionality of the data above the number of input variables, allowing more complex differentiation of the two classes. SVMs can separate two classes at a time and train through supervised learning. An exception is one-class support vector machine (OCSVM), which trains via unsupervised learning. Rather than drawing a barrier between two classes, the OCSVM draws a boundary around a single data class. The model output represents the likelihood of a new point belonging to that one class. Lower values of the output suggest outliers, making this another type of anomaly detection. For example, one study found the ability to detect pathologic aortic dilation using four measurements of this vessel from an echocardiogram. A patient with bicuspid aortic valve or Marfan syndrome may fall out of the normal range for a set of aortic measurements [24,25,26]. The authors argue that such an ML model avoids assumptions typically made about the relationship between biological measurements and patient body size. Such concepts are helpful for detecting disease—potentially earlier and with more clinical significance—based on clinical testing.

### 1.5. What Does Agentic AI Do? How Does the RAG Framework Improve Large Language Models?

**Figure 4:**
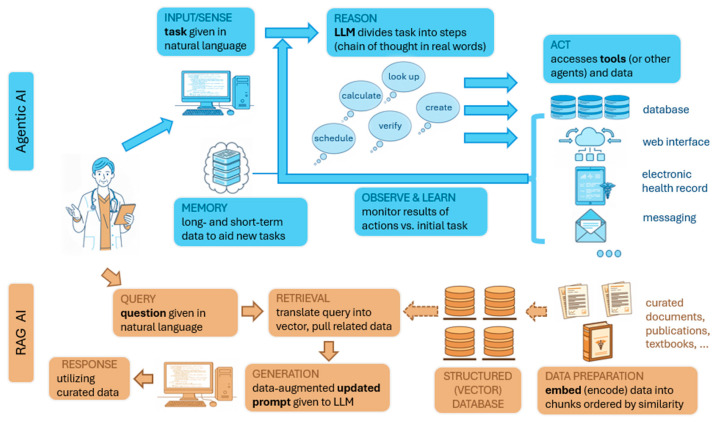
Diagrammatic illustration of agentic AI and retrieval-augmented generation (RAG). Agentic AI applications have flexibility to achieve complex tasks through use of LLMs and technology to access resources and tools autonomously. A RAG system also utilizes an LLM, although it serves the purpose of reducing errors and AI hallucination (inappropriate responses) by drawing upon data that is curated for specific purposes (e.g., contextually created for a specific topic or subspecialty).

Many AI models are what is termed “reactive.” They generate outputs such as predictions, text, or images at the behest of a prompt. Agentic AI builds upon this to solve more complex goals that require multiple discrete intermediate steps and autonomy to take actions or access additional information. Multi-step reasoning and integration of real-time data are obstacles for most LLMs. A key tool to agentic LLMs is ReAct, a portmanteau of “reasoning” and “action” [27]. This method implements a framework that repeats a “thought-action-observation” loop (Figure 4). Each step is framed in human language and then submitted back to the LLM. First, an LLM generates internal reasoning about how to solve the goal (thought). That thought generates a request for additional information, such as a database query or web search (action), which produces a result (observation) that is reintegrated into the next loop of reasoning. As an example, when prompted with the question: “What medication changes should I consider for my patient who is found to have significant QT prolongation on EKG?” The model proposes that it must ask first what medications the patient is taking, then ask which medications prolong the QT interval. This requires accessing the patient’s electronic chart and then a reference for QT-prolonging medications (e.g., crediblemeds.org). The agent determines that the patient is taking azithromycin for mycoplasma pneumonia and that this medication poses a risk for the arrhythmia, Torsades de Pointes. The agent then asks which options for a change to the medication are available. A second search finds that doxycycline can effectively treat mycoplasma without the same risk for arrhythmia. As an error checking step, the model additionally references a clinical decision support online resource that finds a recommendation to employ doxycycline in patients with long QT provided that they are not pregnant. The results are synthesized into a response, including (if desired) reasoning steps. Given the proper interface, such an agent could additionally complete tasks (e.g., ordering a change in the medication after careful review by the provider).

Another technique built upon the basis of LLMs is human curation of its reference data, rather than relying upon the static general knowledge built into the LLM learned from training on mostly non-medical datasets. Reference data may be changed or appended without having to retrain the LLM itself. LLMs gain access to additional resources through **Retrieval-Augmented Generation (RAG)**, a framework allowing models to access resources beyond what exist within the model itself. Data from sources such as scientific papers, published guidelines, textbooks, etc., are embedded (encoded in a specific format) into a database of arbitrary size. RAG queries are first translated into the encoding format of the database. Related data (matching the encoded query) is extracted and provided to an LLM as a new data-augmented prompt. For example, a cardiologist might ask “does my patient with bicuspid aortic valve and aortic measurements of X and family history of Y require surgical aortic repair?” A RAG model linked to consensus statement finds that, lacking specific high-risk criteria, the aortic dimension does not currently meet criteria for root replacement. Researchers using radiology society guidelines and other published resources have demonstrated that RAG-based LLMs can outperform traditional LLMs (and even radiologists) on clinical radiological questions, such as bone fracture classification [28].

### 1.6. What Does a Digital Twin Simulation Accomplish?

A **digital twin** is a virtual model created to simulate a patient, human organ, or medical system. In the case of pediatric cardiology, for example, a digital twin might simulate hemodynamics of a patient’s heart and vasculature or their future clinical course (e.g., after surgical repair of congenital heart disease). Over time, the virtual twin incorporates new data from the physical twin (e.g., from wearables, imaging such as an echo, laboratory/genetic data, etc.), to improve the quality of the simulation. The output of these models may guide clinical decision making (e.g., type of surgical approach to cardiac repair) or foresee potential clinical deterioration (e.g., a patient recovering from surgery in the cardiac ICU or an individual with heart failure being followed as an outpatient). Additionally, digital twins may aid in education and counseling, such as individualized learning experiences for trainees and simulation of patient interactions [1,29,30,31,32]. A current example is the artificial pancreas system for patients with type 1 diabetes, which gathers real time data to create a dynamic representation of the patient, enabling personalized adaptation of care [33]. Kovatchev et al. reported improved clinical outcomes with this method in a randomized control trial (that included some patients < 21 years of age) [34].

## 2. Optimization, Evaluation, and the “Art” of Machine Learning

The success of a machine learning model depends highly on its design and optimization. Design begins before any data is collected, when investigators determine the goal and output of the model. Investigators must estimate whether sufficient quantity and quality of data is available to address the goal successfully. In general, more data examples are better, presuming they adequately represent future, unseen data. For example, a model built to predict left ventricular dysfunction on echo by ECG requires an ample quantity of paired ECGs and echoes. One must also consider quality: e.g., the reliability of echo measurement and the clinical relevance of the degree of predicted dysfunction. In the absence of existing high-quality databases, data collection and processing represent a significant burden in the creation of medical ML models.

Once data is collected, although prior to final training, the model structure and variables that guide training (called hyperparameters) are adjusted to optimize model performance. The structure of a model includes model type (convolutional, recurrent, transformer, etc.), data input size, number of layers, numbers of filters per layer (in CNN models), the type of output(s), and the **cost function** (also known as., loss function) of the model output. A cost function gauges the disagreement between the model prediction and expected result. Most machine learning models learn by minimizing the cost function value through updates to the model parameters, a process called gradient descent. Picture a landscape representing all possible values of an ML model parameters; the height of the hills and valleys represent the degree of inaccuracy. Gradient descent is like steering the model down to the lowest point of error. Unlike parameters (weight, biases) that models learn through training, **hyperparameters** refer to variables set by a data scientist that control the training of the model (steering it, if you will). For example, this includes learning rate (e.g., how fast the parameters are modified each epoch), batch size (the number of training examples input into the model before updating the parameters), regularizers (techniques to prevent model overfitting and unstable model training), and an initialization strategy (the method of randomly setting the model parameters before training starts). Additionally, one chooses an **optimizer**: a methodology for how to update model parameters efficiently during training based on the cost function.

Data scientists often speed up training of new models by “recycling” older models in a process called **transfer learning**. Rather than starting in a random state, a new model designed to analyze ultrasound images may start from an existing model previously trained to identify animal species, for example. This technique works by “transferring” the ability to extract basic features to the new model that may seek a quite different goal. The need to recognize lines, textures, and basic image patterns is shared between identifying a type of dog and detecting a dilated kidney. Some of the initial model layers are often kept static (frozen) during retraining, facilitating rapid development of the new model. This technique reduces the required amount of training data, training time, and computational power compared with starting from nothing. Indeed, self-supervised learning and use of pretext tasks (see Section 1.2) is a form of transfer learning, where models such as LLMs acquire the basics of language prior to being trained as functional chatbots.

In supervised learning, investigators often split data into **training** and **validation** cohorts for model optimization (e.g., 80%, and 20%, respectively). Unsurprisingly, the training set is the content from which the model learns. The validation set then helps to tune the model. After training with a given set of hyperparameters, a model is evaluated on this validation cohort—being a representative sample of the full data set. Hyperparameters are adjusted systematically until researchers select an optimal model. Often, the simplest model with the best performance metric on the validation set is chosen. Subsequently, investigators will evaluate an ML model on a test cohort drawn separately from the training and validation cohorts, either from the same population or—ideally—derived from a separate population entirely (e.g., another medical center). Note that the term “validation” may sometimes be applied to the testing of fully trained models. For example, a model is “externally validated” when evaluated on examples from location(s) outside of the main research site, supporting the strength and applicability of the model.

Various metrics may be used to compare the performance of predictive models. For classification models, common choices include area under the receiver operator curve **(AUC)** or **F1-score** (a single metric incorporating both sensitivity and positive predictive value). For regression models or those with outcomes that are continuous values, researchers may choose metrics such as root mean squared error (RMSE). Other options for performance metrics are available. Choice of the metric depends on the outcome (e.g., single class vs. multiple class) and whether there is significant difference in prevalence of different predicted outcomes. Much of ML optimization seeks to balance the fit of a model such that it trains enough to make accurate predictions and generalizes enough to be applicable to novel data. Overly simple or undertrained models make poor predictions on both training and validation sets, a situation called **underfitting**. Conversely, performing well on training data yet poorly on a validation cohort results from **overfitting**, potentially due to an overly complex model that “learns” the training data. While various techniques can mitigate these issues, a common solution is simply to train a model with more high-quality training data.

## 3. Understanding What Machine Learning Models Are Doing

It is only human to desire an inner understanding of machine learning models. Due to their level of complexity (enormous size and degree of non-linearity), forcing transparency on the mechanics can be elusive for these black box-like tools. However, approaches toward explainability do exist. **Saliency mapping** attempts to highlight the components of the input that are most important for the model to arrive at a decision. One example is Grad-CAM (mathematically tracing the most influential factors backwards from the output of the model layer-by-layer until reaching the input layer). One might also repeatedly alter part of the input to see which input features are most critical to the prediction; this is called perturbation analysis. Saliency maps can help confirm that a model is “looking at” features that makes sense and may aid in academic understanding [4,8]. Other ML models inherently let us know “what they are looking at” from the input via object localization. Famous for its contribution to self-driving technology to identify other cars, signs, stoplights etc., object localization can also highlight specific cardiac structures from an echocardiogram or coronary artery disease from angiography, for example [35,36].

## 4. Collaboration on the Training of Medical ML Models

**Figure 5:**
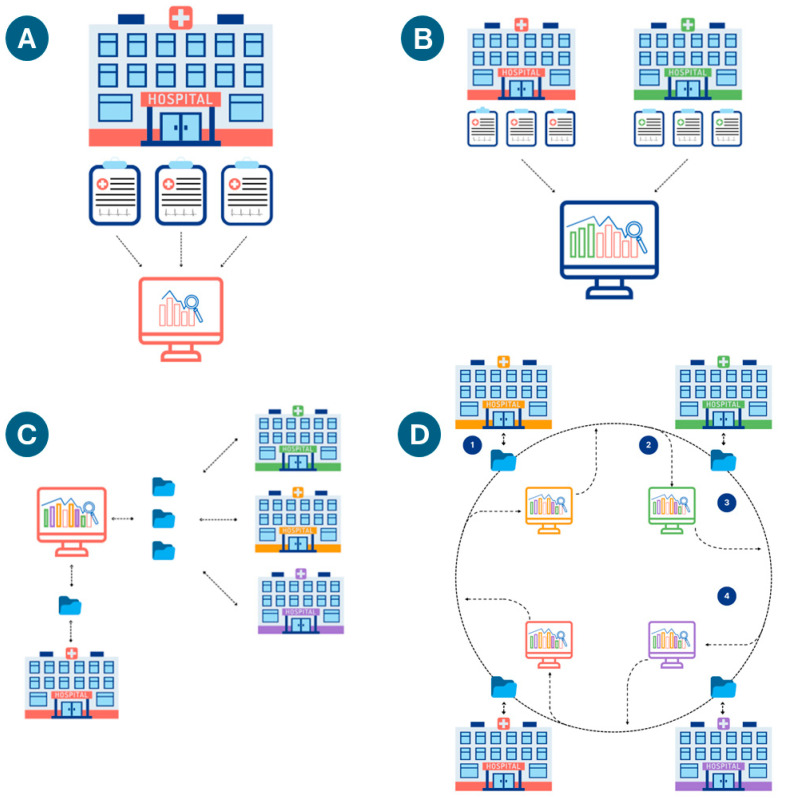
(**A**) Local Learning: a model at a single institution trains on a local server with local data. (**B**) Central Learning: models train on data from multiple institutions on a centralized or cloud server (data is sent out of each center). (**C**) Federated Learning: training occurs at each institution, with periodic updating of a centralized model on a shared server, which redistributes updated copies of the updated algorithm to all the institutions. Local data remains at each institution; only the model parameters sent back and forth from the main server. (**D**) Swarm Learning. An initial base model is created. (1) Each institution trains the model with local data; (2) Intermittently, the updated model parameters are aggregated at a designated local server; (3) The updated model gets sent back to all centers; (4) The process is iterated until a stopping condition is met.

Several challenges limit the creation of machine learning models, particularly in pediatric medicine. Individual medical centers may lack sufficient data to train large ML models, particularly for rare diseases. Rare diseases are more common in pediatrics and may result in greater financial cost than in adults [5,37]. Most individual varieties of congenital heart disease are rare; for example, tetralogy of Fallot (the most common type of cyanotic congenital heart disease) affects less than one in every 2000 U.S. newborns [38]. Rarity limits the benefit of **local learning**, where models train in situ on local data. Such models may be at risk for both underfitting and for bias due to local underrepresentation of specific diseases, treatments, or patient demographics, for example. Validating these models on external data (e.g., patients/data from another medical center) may support their accuracy and generalizability. Alternatively, data from multiple centers may be combined in **central learning**, although sharing data with other centers may require significant investment in data deidentification and efforts to meet national and institutional regulations. Potential workarounds for collaborative model creation include **Federated learning** and **Swarm learning**. Federated learning trains a single model by distributing copies to each site. Local data stays on local servers. Each site trains its local model for a given period, after which all participants update the central model. The updated model is downloaded by each site, and this process continues until training is complete. The principal goal of federated learning is data privacy. **Edge computing** is a similar concept, in which raw data is processed on distributed nodes (often initially processed locally)—although with the goal of reducing data transfer and demands on centralized computation. While data privacy may be increased in some cases, this is not the primary aim or a necessary result of this technique. **Swarm learning** typically works with edge computing to decentralize all computations. As with federated learning, models are trained locally (on each node) for a given period. Rather than relying on a centralized model, however, training is combined at one of the nodes—dynamically chosen by the group—that updates the parameters and redistributes the updated model (Figure 5) [1,39].

## 5. Hurdles in the Implementation of Medical AI Applications

Beyond the challenges to successful model creation noted above (paucity of data in rare diseases, necessity of collaborative research), additional barriers may impede the progress of AI toward pediatric clinical practice. One barrier is effective funding and resources. Published data has revealed a disparity in sponsorship and grant allocation for pediatric versus adult medical research [40,41]. Machine learning research requires storage and computation on large amounts of data—potentially terabytes (trillions of bytes) in size in the case of medical imaging data such as echocardiograms. Machine learning training is performed on cloud platforms or specialized hardware, requires support of data scientist expertise, and may incur additional costs for data de-identification. Even prior to data collection, researchers must also ensure ethical stewardship of patient data and privacy, which increases significantly in complexity when data is shared between medical centers or with industry. Agreement over intellectual property must also be settled prior to any collaboration, necessitating harmonization between medical, legal, research, and medical leadership teams. Furthermore, significant heterogeneity in data formatting and quality may be present—either from one medical center to another or over different eras of record keeping [42]. Different equipment or equipment settings (e.g., echo machine or ECG acquisition frequency) may contribute to data heterogeneity, placing additional demands on models to be adequately generalizable across different medical practices. Ideally, training sources will include annotated or coded data (e.g., echoes that have formatted reporting and measurements). Unfortunately, some data may only be stored as text or even scanned images; such cases may require the addition of preprocessing data, such as with natural language processing (see above), or otherwise tedious manual effort to properly encode data.

In the U.S., the Food and Drug Administration (FDA) regulates clinical application of both ML software and AI/ML-enabled medical devices. The FDA term for standalone software, such as predictive models that function independently of medical equipment, is “software as a medical device.” As of writing, 950 AI devices have been approved since 1995 (the majority 75% in the field of radiology, followed by 11% in cardiology) [43]. The FDA also publishes guidelines on best practices in AI and ML devices/software [44]. The FDA approves new or modified medical devices via 510(k) clearance, which reviews the safety and efficacy of devices that are neither implanted nor able to injure a patient directly if malfunctioning [45]. In Europe, AI models require CE (“Conformité Européenne”) designation to indicate that the devices meet safety, health, and environmental protection requirements. In addition, a European Union law, called the General Data Protection Regulation (GDPR), governs the usage of personal data to protect individual privacy and autonomy. In China, the National Medical Products Administration (NMPA) guides and supervises the development of AI technologies in healthcare, alongside other medical devices and drugs. Other international regulatory agencies include the International Organization for Standardization (ISO), the World Health Organization (WHO), and the International Electrotechnical Commission (IEC), which provide global guidelines on standards for AI applications in health care [46].

Even when approved by regulators, AI technology must be vetted by potential clinical adopters who need to consider site-specific cost–benefit analysis and risk assessment. The coordination of technology integration into the medical system, including the electronic health record (EHR), must be addressed with the aid of information services and clinical support staff. Finally, education on and socialization of AI tools requires time and dedicated clinical allocation. Moreover, clinicians—especially those early in their careers without a lot of experience—must understand the limits of assistive and predictive models to avoid the risk of overreliance on these technologies, trusting them above sound clinical judgement, a fallacy called **automation bias** [1,3,47,48].

## 6. Current and Future Directions for AI in Pediatric Cardiology

At the time of this article, over 100 AI-based technologies specific to cardiovascular care have met the FDA safety and effectiveness standards and are already in use in some centers. Most were developed primarily for adult patients, leaving a gap in the application of AI in the pediatric space [43]. While some AI tools originally trained with adult data have been explored for pediatric use, this tactic raises several issues. Most adult data lack sufficient content pertaining to congenital malformations. Further, children have differences in organ makeup (e.g., fat content) from adults and exhibit significant differences in the incidence and presentation of acquired diseases [49]. Commercially available smart stethoscopes by brands like Eko and Stethaid offer AI detection and classification of heart sounds including murmurs from children with congenital heart disease. Some of these have been validated in pediatric populations and show a high percentage of agreement when compared to physician auscultation and echocardiogram findings. At this time, Eko stethoscopes are FDA cleared, while Stethaid stethoscopes are not [34,35]. Other efforts in this area include the work of Thompson et al., who validated an AI algorithm that accurately classify pathologic and normal heart sounds based on a database [50,51,52].

A machine learning classifier (MLCs) AI system tested by Liang et al. was able to query thousands of data points from the EHR and was as accurate as experienced pediatricians in diagnosing common childhood illnesses across multiple organ systems [53,54]. MLCs offer the ability to summarize patient history and test results, pre-write clinical notes based on spoken conversation, simplify billing procedures, and even assist in patient scheduling. Most of these models are not widely applied in clinical practice in pediatrics, yet.

A current example of MLC already used in some centers is “ambient note” platforms. These automate clinical documentation from audio captured during patient encounters, allowing for more personalized patient engagement and streamlining workflows, potentially reducing physician burnout. There are currently at least 90 commercially available options, none of which are FDA cleared. The lack of regulatory guidelines for these products, as well as concerns regarding equity, interoperability, and transparency are possible limiting factors for clinical implementation of some of these products [55]. A recent cross-sectional survey of 43 major US health systems found that “Ambient Notes” was the only AI use case boasting 100% of respondent organizations reporting some level of adoption. However, survey findings did not differentiate between pediatric and adult providers [56].

There are hundreds of commercially available wearable technologies marketed for health monitoring, but only a subset has obtained FDA approval for specific conditions [57]. For example, the Owlet sock is a monitor sock that is currently FDA cleared for pulse oximetry in infants, allowing for at home non-invasive monitoring of patients with SVT. Newer versions of the Apple watch have FDA clearance for ML rhythm classification, and can detect signs of atrial fibrillation. Also, a ML algorithm used by Zio Patch (a wearable heart rhythm monitor) can create reportedly expert-level preliminary rhythm reports, generate alarms, and route rhythm strips [57,58]. However, validated data on these devices is limited at this point [59,60].

Further directions for AI in pediatrics are virtually limitless. We encourage the reader to explore prior publications discussing the use of AI to improve interpretation and performance of the pediatric ECG [61], pediatric and fetal echo [62], and pediatric cardiac MRI [63]. The authors speculate that the success of self-supervised learning and transformer models (see above) in large language models may likewise revolutionize medical imaging such as echocardiography. The powerful ability of this approach to learn from large scale data without the requirement for exhaustive labeling by experts holds tantalizing promise.

AI may also benefit subspecialized areas within pediatric cardiology. The cardiac intensive care unit cares for post-operative and severely ill patients. ML-based early detection of adverse events based on vital signs, hemodynamic, laboratory, and other clinical data might decrease morbidity and mortality. In a similar vein, ML augmentation of patients at home with wearable medical devices may reduce hospitalization time by providing early detection of cardiovascular decompensation.

Procedural medicine may benefit from personalized digital twinning. Cardiac surgical planning for patients with complex congenital heart disease represents a formidable challenge. Simulation of cardiothoracic surgical outcomes may allow providers to choose the most ideal timing and technique of the repair, using a combination of patient-specific imaging, other clinical data, and principles of hemodynamics. Interventions in the cardiac catheterization and electrophysiology labs may also benefit from pre-procedural simulation utilizing the same data, potentially decreasing adverse outcomes, radiation exposure, and time under anesthesia.

## 7. An Approach to Evaluating an AI Model: Essential Questions

A thorough assessment of a model can help assess the suitability of the AI solution for specific needs. Essential considerations include:

☑ Purpose and Scope: Does the model clearly define its goal and relevance?
☑ Actionability: What will be done with the output? The Outcome-Action Pairing (OAP) can help frame the effects of implementation:
  - Outcome: The purpose of the model (risk stratification, diagnosis, etc.)
  - Action: The intended step to be taken considering the outcome (e.g., treatment or further testing based on diagnosis, appointment with a primary physician to avoid readmission, etc.). Consider how this step will be taken.
☑ Data Quality:
  - Was the algorithm trained on data representative of the population to which it will be applied (age range, sex, disease prevalence, etc.)? Might differences between the training and target populations bias the output?
  - Was the data correctly labeled in the case of supervised learning? What defined the ground truth? Review the key terms and how they were defined.
  - Was the data complete? How were incomplete data/missing values addressed?
  - With what volume of data was the model trained? Is the data balanced (for example, a similar number of samples for each category in a classification model)? How were any class imbalances addressed?
  - Are all important variables associated with the main outcome represented in the data?
☑ Model Performance: Evaluate performance metrics (accuracy, sensitivity, specificity, ROC curves, AUC, F1 scores, etc.). Check for independent validation and evidence of generalizability. Was the training data separate from validation and test cohorts? Are any unfair hints toward the outcome given to the model in training that would not be present in real-world application (called “leakage”)?
☑ Clinical Integration: how does (or would) the model integrate into clinical workflows and EMR systems? Who would be interpreting the output of the AI models and how does this translate to patient care? Could it have any unintended consequences if misused?
☑ Safety and Ethical Implications:

Might implementation lead to biased or unfair treatment?

- Consider subgroups of populations: Would distinct groups have different outcomes? Would implementing the model impact people of certain socio-economic groups, ages, sex, etc., differently?
- Will models run externally from sites of clinical practice? Evaluate data privacy compliance and potential cybersecurity risks.

☑ Regulations and Compliance: Verify regulatory approvals or certifications (e.g., FDA, “Conformité Européenne”/CE).
☑ If a model has already been implemented: Assess ongoing performance and updates, incorporation of new data, and the effect on patient outcomes.

When evaluating an AI tool for potential implementation, consider evaluating it alongside those interdisciplinary team members interacting and affected by the tool. Review as a team to answer if and how the tool should be incorporated in a particular setting (physicians, nurses, administration, legal, risk management, information services, etc.) [44,64].

## 8. Conclusions

AI has already steered our relationship with medical data, yet the opportunity for improvement and integration in pediatrics is substantial [1,65]. Medical centers generate data on a massive scale through examination, testing, imaging, and medical documentation. Successful AI thrives on big data, offering efficient solutions to many realms of patient care. However, pediatric AI applications lag behind those in the more extensively funded adult medical space. Pediatric providers require a grasp of fundamental AI principles to enable the success of ML models and AI-enabled devices. Expansion of AI education content into pediatric and subspecialty training programs will also become imperative. When evaluating an ML device, consider its initial goal, what data was used to create it, how it was optimized and tested, and how you will apply its output while avoiding automation bias. Collaborating in multidisciplinary teams including providers, computer scientists, other clinical stakeholders, and IT experts can facilitate the safe integration and scaling of clinically relevant technology. For those seeking to engage in pediatric AI research, multisite cooperation will be the norm to ensure robust and validated products. Soon, AI tools may become ubiquitous in general and subspecialty pediatric care. If adopted judiciously, such tools offer the potential to improve efficiency, increase disease detection, aid interventional planning, and enhance long-term outcomes.

## Data Availability

Not applicable.
